# Fabrication of Scaffold-Based 3D Magnetic Nanowires for Domain Wall Applications

**DOI:** 10.3390/nano8070483

**Published:** 2018-06-30

**Authors:** Dédalo Sanz-Hernández, Ruben F. Hamans, Johannes Osterrieth, Jung-Wei Liao, Luka Skoric, Jason D. Fowlkes, Philip D. Rack, Anna Lippert, Steven F. Lee, Reinoud Lavrijsen, Amalio Fernández-Pacheco

**Affiliations:** 1 Cavendish Laboratory, University of Cambridge, JJ Thomson Avenue, Cambridge CB3 0HE, UK; dsh41@cam.ac.uk (D.S.-H.); jwl41@cam.ac.uk (J.-W.L.); ls604@cam.ac.uk (L.S.); 2 Department of Applied Physics, Eindhoven University of Technology, P.O. Box 513, 5600 MB Eindhoven, The Netherlands; r.f.hamans@student.tue.nl (R.F.H.); r.lavrijsen@tue.nl (R.L.); 3 Department of Chemistry, University of Cambridge, Lensfield Road, Cambridge CB2 1EW, UK; jwmo2@cam.ac.uk (J.O.); ahl29@cam.ac.uk (A.L.); sl591@cam.ac.uk (S.F.L.); 4 Center for Nanophase Materials Sciences, Oak Ridge National Laboratory, Oak Ridge, TN 37831, USA; fowlkesjd@ornl.gov (J.D.F.); prack@utk.edu (P.D.R.); 5 Materials Science and Engineering Department and Bredesen Center for Interdisciplinary Research, The University of Tennessee, Knoxville, TN 37996, USA

**Keywords:** 3D-nanoprinting, Focused Electron Beam Induced Deposition, nanomagnetism, FEBID, nanowire, nanofabrication, direct write, thin film

## Abstract

Three-dimensional magnetic nanostructures hold great potential to revolutionize information technologies and to enable the study of novel physical phenomena. In this work, we describe a hybrid nanofabrication process combining bottom-up 3D nano-printing and top-down thin film deposition, which leads to the fabrication of complex magnetic nanostructures suitable for the study of new 3D magnetic effects. First, a non-magnetic 3D scaffold is nano-printed using Focused Electron Beam Induced Deposition; then a thin film magnetic material is thermally evaporated onto the scaffold, leading to a functional 3D magnetic nanostructure. Scaffold geometries are extended beyond recently developed single-segment geometries by introducing a dual-pitch patterning strategy. Additionally, by tilting the substrate during growth, low-angle segments can be patterned, circumventing a major limitation of this nano-printing process; this is demonstrated by the fabrication of ‘staircase’ nanostructures with segments parallel to the substrate. The suitability of nano-printed scaffolds to support thermally evaporated thin films is discussed, outlining the importance of including supporting pillars to prevent deformation during the evaporation process. Employing this set of methods, a set of nanostructures tailored to precisely match a dark-field magneto-optical magnetometer have been fabricated and characterized. This work demonstrates the versatility of this hybrid technique and the interesting magnetic properties of the nanostructures produced, opening a promising route for the development of new 3D devices for applications and fundamental studies.

## 1. Introduction

**Figure 1:**
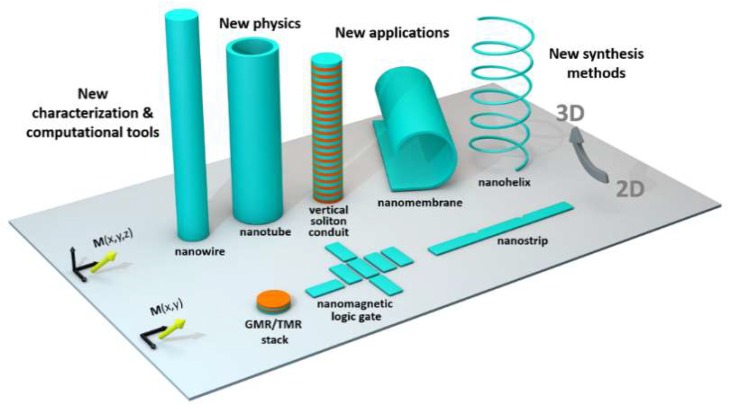
Transitioning from two-dimensional (2D) to three-dimensional (3D) nanomagnetic systems requires new synthesis methods, new characterization techniques, and new computational tools. This transition opens a new set of possible applications, as well as new physical phenomena arising from the interplay between 3D properties and magnetization at the nanoscale. Typical nanostructure scales (e.g., nanowire/nanotube diameters, 2D nanostrip widths, curvature radii of nanohelices and nanomembranes) are 30–500 nm, i.e., a few times a characteristic magnetic length of the system such as the dipolar exchange length.

During the last decades, nanopatterned magnetic materials have played a fundamental role in our society—enabling the development of ever-smaller hard disk media and bringing to unprecedented levels our ability to share information. Today, going beyond hard disk media, magnetic nanodevices offer key advantages such as non-volatility and low power consumption, with technologies such as Spin-Transfer Torque Magnetic Random-Access Memory (STT-MRAM) close to large-scale production [1]. Based on this success and the great advances in the synthesis, characterization and modelling of three-dimensional magnetic nanomaterials over the last years [2], it is now possible to think beyond two-dimensional patterning: Fully three-dimensional systems start to become possible. In these systems, the interplay between magnetization and 3D properties results in novel physical phenomena. This opens up new routes for applications and enables, amongst other benefits, much higher device densities through vertical stacking [2]. Figure 1 illustrates this change in paradigm, comparing three planar structures (Giant Magneto Resistive (GMR)/Tunneling Magneto Resistive (TMR) multilayer stack, nanomagnetic logic gate, and nanostrip) to a series of 3D structures (nanowire, nanotube, vertical soliton conduit, nanomembrane and nanohelix), outlining the need for new synthesis, characterization and modelling methods for this paradigm shift to take place.

One of the key technical breakthroughs required to enable this 3D transition is the development of synthesis methods that can controllably sculpt magnetic materials at the nanoscale. Successful approaches to 3D nanopatterning of magnetic materials include [2]: Rolled-up nanotechnology [3], chemical deposition onto 3D templates [4], physical vapor deposition onto self-assembled [5] and optically written [6,7] scaffolds, and direct 3D nano-printing of magnetic materials by Focused Electron Beam Induced Deposition (FEBID) [8]. Amongst these methods, 3D nano-printing presents unparalleled flexibility for rapid prototyping at the nanoscale in terms of geometry and resolution. Recent progress in modelling [9] and Computer Aided Design (CAD) tools [10] make it possible to nano-print intricate 3D segment networks in a highly controlled manner.

In this work, we describe in detail recently developed methods [11] to expand the capabilities of 3D nano-printing using FEBID [10]:

- Nanostructure width (segments become flat nanowires) is achieved by using a dual-pitch pattering strategy.
- The catalogue of accessible materials is expanded by combining 3D nano-printing with physical deposition methods.
- Growth limitations for low segment-angle structures are overcome by employing substrate tilting during growth.

The combination of these methods greatly increases the types of nanostructures available through 3D nano-printing and the range of applicability of the technique.

In what follows, we will discuss our approach used to introduce width, the importance of nanostructure supports during thin-film deposition, and how substrate tilt is used to create suspended ‘staircase’ nanostructures in which some segments lie parallel to the substrate. Following this discussion, a practical case in which individual nanowire devices have been tailored to match a dark-field magneto-optical setup will be presented. This precise tailoring employs all of the above techniques and has made it possible to study and control information transfer between a 2D film and a 3D nanowire for the first time [11].

## 2. Materials and Methods

In this work we combine 3D-nanoprinting of non-magnetic scaffolds using FEBID with thermal evaporation of a magnetic thin film of Permalloy (Ni_80_Fe_20_) onto the scaffold. The precursor used for scaffold nano-printing was Trimethyl (methylcyclopentadienyl) platinum—(CH_3_)_3_Pt(CpCH_3_), which results in Pt-C compounds upon electron irradiation, with typical Pt contents being between 10 and 20% [12]. Parts 2.1 and 2.2 of this section describe the nano-printing process, with part 2.3 describing the thermal evaporation step.

### 2.1. 3D-Nanoprinting by Focused Electron Beam Induced Deposition (FEBID)

**Figure 2:**
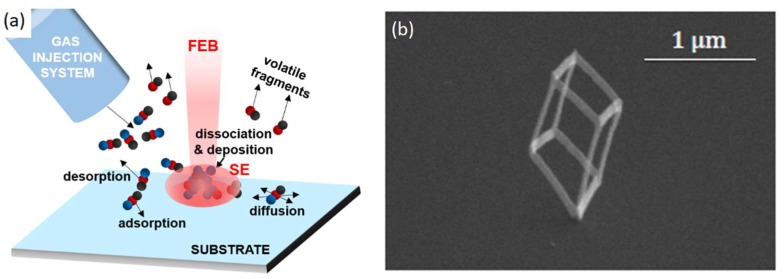
(**a**) Physical processes in Focused Electron Beam Induced Deposition (FEBID). A precursor gas is injected closely to the impact region of a Focused Electron Beam (FEB). Precursor molecules are adsorbed onto the substrate, where they are dissociated upon Secondary Electron (SE) impact, releasing volatile fragments and leaving a solid deposit on the substrate; (**b**) Example of a freestanding three-dimensional cube nano-printed using FEBID. The cube contacts the substrate only at its lowest corner.

Focused Electron Beam Induced Deposition (FEBID) [12,13] is a bottom-up maskless lithography technique for 2D and 3D nanostructures that employs a highly focused beam of electrons, typically between 1 and 30 keV in energy, to locally dissociate a precursor gas. Several gases are available, allowing deposition of different metals and insulators [12]. The highly focused beam of electrons is provided by a Scanning Electron Microscope (SEM), and gas is injected closely (50 to 300 µm) to the beam impact region using a fine nozzle (50 to 800 µm in diameter). Experiments take place in vacuum in an SEM chamber. The physical processes involved in FEBID are outlined in Figure 2a: After injection, molecules travel towards the substrate, where they adsorb, diffuse and desorb at different rates [9]. When impacted by the Focused Electron Beam (FEB) and its associated Secondary Electrons (SE), precursor molecules dissociate, releasing volatile fragments and leaving a solid deposit on the substrate. Repeated exposure on the same spot leads to a build-up of material and results in three-dimensional growth. The precise and computerized control over FEB positioning intrinsic to SEMs enables the geometry of the resulting 3D deposit may be precisely controlled. When combined with system calibration and advanced CAD tools [10], this precise beam control allows the user to create complex, freestanding, 3D nanostructures (See Figure 2b).

### 2.2. FEBID Parameters and Calibration

Experiments were carried out using a field emission gun SEM, with a typical base pressure of ~10^−6^ mbar, and pressures during growth of ~7 × 10^−6^ mbar. Acceleration voltages of 30 keV have been used, with beam currents of 25 pA.

**Figure 3:**
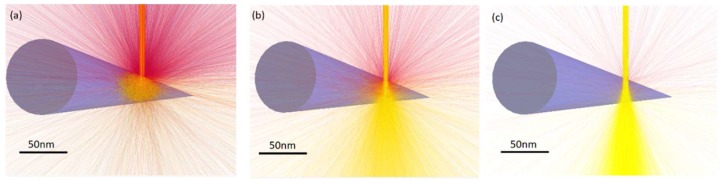
Simulated primary and secondary electron trajectories at different beam acceleration voltages upon incidence of a 5 nm beam on a horizontal Pt cone. (**a**) 3 keV; (**b**) 10 keV; (**c**) 30 keV. The best resolution is obtained at high acceleration voltages, since the low interaction between beam and cone leads to a higher spatial confinement of the electron irradiated region. All trajectories are colored by energy loss, increasing from yellow to red and blue.

Of the great number of parameters affecting FEBID nano-printing [12,13,14], the incident electron energy (SEM acceleration voltage) is key to the resolution of the process [15]. This is illustrated in Figure 3. Monte Carlo electron trajectories have been simulated using CASINO [16] for a 5 nm beam radius and acceleration of 3, 10 and 30 keV respectively. The beam impacts a Platinum tip transversely.

At low acceleration voltage (Figure 3a) many electrons lose energy and are backscattered by the Pt, leading to a large volume of space where decomposition of precursor may occur (leading to low resolution 3D-printing). On the contrary, at high acceleration voltage (Figure 3c) fewer electrons lose energy or are scattered, leading to a better spatial confinement of the electron distribution, and therefore to a higher resolution nano-printing process. The confinement of the electron distribution is further improved by inherently smaller SEM beam spots at high voltage.

**Figure 4:**
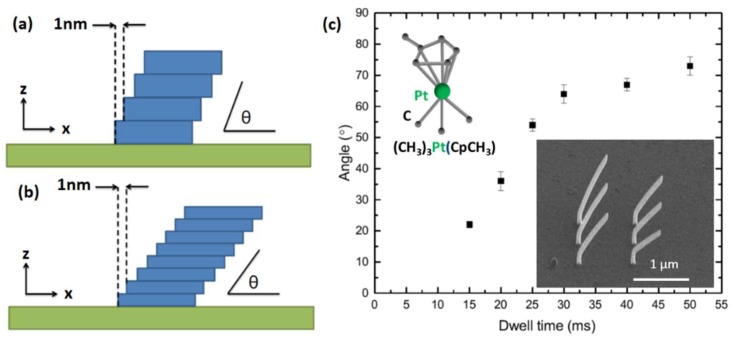
Calibration Experiment. (**a**,**b**) Deposits resulting after scanning the electron beam with a 1 nm pitch, with long (**a**) versus short (**b**) dwell times. A long dwell time (**a**) results in more electron exposure and higher vertical growth per dwell spot, giving a higher angle θ; (**c**) Angle θ is measured as a function of dwell time for a constant pitch of 1 nm, resulting in a set of calibration points. Insets: Chemical structure of the precursor used and set of nanowires used to obtain the calibration points. For all wires, a small vertical pillar has been grown prior to the segment used for calibration, to facilitate the segment lifting from the substrate.

Precise 3D nano-printing relies on system calibration prior to every fabrication session [10,14]. This calibration can be encapsulated by a single experiment in which the electron beam is scanned over the substrate, in a straight line, taking discrete steps of 1 nm ‘pitch’. After each step the beam is kept stationary for a certain ‘dwell time’, triggering vertical growth. The ratio between pitch and vertical growth results in an angle θ which can be measured from SEM images. Figure 4a,b illustrate this process: In Figure 4a a long dwell time is used and a large vertical growth occurs at each step, resulting in a large segment angle. In Figure 4b a shorter dwell time is used and the angle of the segment is smaller.

During a calibration experiment segment angles are measured as a function of dwell time (Figure 4c) and a curve is fitted to the data using either linear interpolation or a 2D surface evolution model [10], making it possible to determine the dwell time required to produce subsequent segments at a given angle to the substrate.

An important feature in the dependence of segment angle with dwell time is the large slope of the curve at low angles: Even small changes in experimental conditions can lead to unwanted deviations in segment angles and this makes it challenging to repeatably fabricate structures with very shallow features. To overcome this issue, modifying the experiment by adding substrate tilt (as described in Section 3.2) can be exploited.

### 2.3. Thermal Evaporation of Magnetic Material

**Figure 5:**
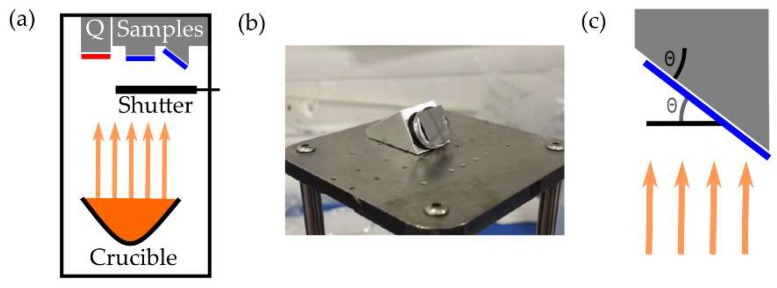
Thermal evaporation of a magnetic material. (**a**) Schematic of thermal evaporator components. A crucible containing Permalloy is heated to deposit a thin film onto the samples and a Quartz crystal microbalance (Q); (**b**) Angled holder used to perform thermal evaporation perpendicular to the nanostructures; (**c**) Schematic of a thermal evaporation process perpendicular to a nanostructure (black), and therefore at an angle θ to the substrate (blue).

The techniques presented above have been employed to fabricate non-magnetic scaffolds at the nanoscale. To provide magnetic functionality, a thin film of Permalloy is thermally evaporated onto the sample. The evaporator (Figure 5a) consists of a vacuum chamber with a Permalloy-containing crucible, a shutter and a quartz crystal microbalance.

After pumping overnight, the chamber is baked at 200 °C for about 10 min to remove any moisture. The pressure is measured using an ion gauge and eventually reaches a value on the order of 10^−^^7^ mbar. The Permalloy is evaporated by applying a ~40 A current to the crucible and the deposited thickness is monitored using the quartz crystal microbalance.

The angle at which Permalloy is deposited onto a structure affects properties such as magnetic coercivity [17]. Thus, in order to optimize the magnetic properties of the structures, it may be desirable to mount the samples such that the evaporation takes place at normal incidence with respect to the nanostructures (Figure 5c), using a holder such as that shown in Figure 5b. Magnetically-active scaffolds could interact with the magnetic thin film via interfacial effects. We do not expect this to be the case for the precursor and conditions used in this study: the high percentage of amorphous carbon of the as-grown material (>80%, see Section 2), and a thin layer of carbon typically present at the surface, as a result of imaging prior to thermal evaporation, will lead to a magnetically-inert interface.

## 3. Results

In this section we first present how FEBID nano-printing has been employed to create scaffolds of variable width, as well as scaffolds with horizontal segments. Then thermal evaporation onto these scaffolds and the role of strain relaxation will be presented, concluding with an example of the nanostructure tuned for dark-field optical magnetometry.

### 3.1. Wide Nanostructures: Dual-Pitch Nanopatterning

So far, 3D nano-printing using FEBID has been mostly focused on the creation of segments with widths determined by the intrinsic resolution of the technique [10,14,18]. Extending this approach to fabrication of wider structures is an important step towards the generalization of 3D nano-printing. Here, we have developed a dual-pitch strategy for this purpose.

**Figure 6:**
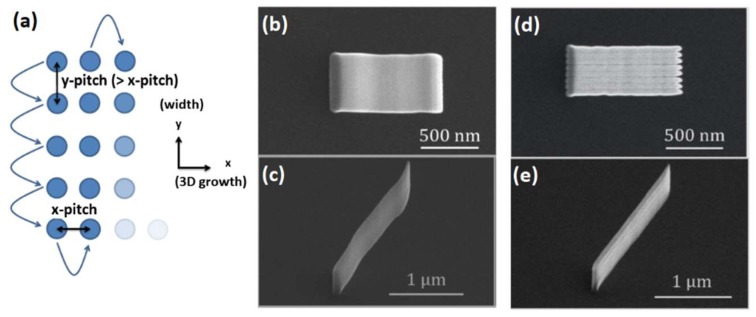
Dual-pitch nanopatterning. (**a**) Defining ‘x’ as the direction of 3D growth, a transverse pitch is defined along the ‘y’ direction in order to create structures with controllable width. The beam scanning strategy after addition of the ‘y’ pitch follows a serpentine pattern starting from the top-left site, as indicated by the arrows; (**b**,**c**) Top and side views of a wide nano-ramp grown using an optimized y-pitch of 30 nm; (**d**,**e**) Top and side views of a wide nano-ramp grown using a y-pitch of 60 nm.

Specifically, we follow a scan strategy based on a serpentine pattern: A standard 1 nm pitch is used along ‘x’ (the direction of 3D growth) and a larger transverse pitch in the ‘y’ direction (See Figure 6a) is used. The serpentine scanning pattern is illustrated in Figure 6a: starting from the top-left pixel, the beam is scanned in the -y direction; when the bottom left pixel is reached, the beam shifts one step in the x direction and repeats the previous y scan, this time in the +y direction. This serpentine pattern is continued for the desired length of the segment along the x direction. As shown in Figure 6b–e, the choice of y-pitch is important in terms of structure morphology and associated roughness. If too large, the wide structure becomes discretized into individual segments (Figure 6d, 60 nm pitch). If too small, the calibration described in the previous section is no longer valid, since the overlap between adjacent points results into a significantly higher dose. For the conditions described above, a y-pitch of 30 nm was found to provide optimum roughness and small deviations of θ-angles with respect to the single-pixel segments (Figure 6b).

**Figure 7:**
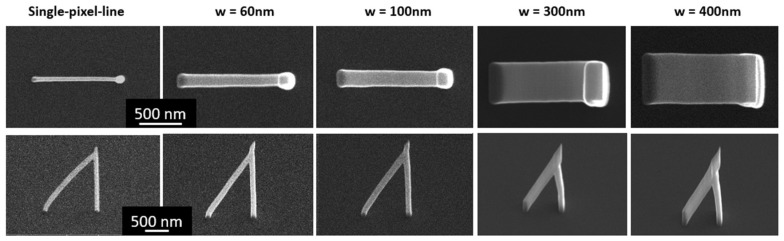
Width control. Top and side views for a set of nanostructures grown using different pattern widths ‘w’.

After choosing the correct pitch in the transverse direction, the total width of the nanostructure may be tuned by choosing a different number of pixels in the transverse direction. Five cases of nanostructures grown at 60° with increasing width ‘w’ are shown in Figure 7. ‘w’ is defined by the beam scanning pattern, with the resulting nanostructure being wider than this value.

### 3.2. Horizontal Nanoprinting Using Substrate Tilt

3D nano-printing at shallow angles is a challenging task: the high susceptibility of angles to small changes in experimental conditions increases drastically as the segment angle is reduced [10]. Furthermore, as in many macroscopic 3D printing techniques, growth in FEBID cannot occur in a completely horizontal manner: some support material is required, rendering a minimum segment angle of ~10 degrees.

**Figure 8:**
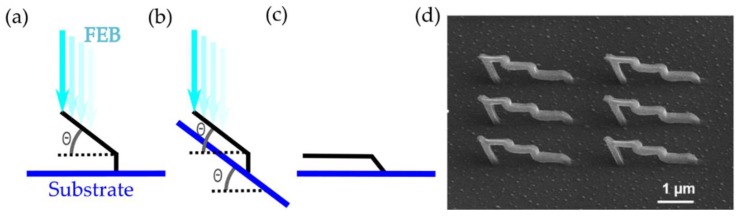
Use of substrate tilt during growth. (**a**) Example nanostructure grown at an angle θ on a flat substrate; (**b**) Nanostructure (**a**) grown using a substrate tilt of θ; (**c**) Result of growing nanostructure (**b**); (**d**) Example of ‘staircase’ nanostructures grown using substrate tilt.

3D nanostructures with segments parallel to the substrate can however be reliably nano-printed by applying a tilt to the substrate. Figure 8a–c illustrate this principle: In Figure 8a, a segment is grown at an angle θ, on top of a small vertical support. The same growth is repeated in Figure 8b, however this time with the substrate tilted by an angle θ with respect to horizontal; this results in a segment parallel to the substrate, as shown in Figure 8c. The application of this principle is exemplified in Figure 8d, where a set of six ‘staircase’ nanostructures have been fabricated.

The same principle of substrate tilting can also be used to improve the repeatability of nanostructures requiring low angles to the substrate (e.g., 20°). Adding a substrate tilt of e.g., 30° results in growth taking place at 50° to the horizontal, which, as discussed in Section 2.2, is not as susceptible to small variations in experimental conditions.

### 3.3. Thin-Film Evaporation onto 3d Scaffolds

**Figure 9:**
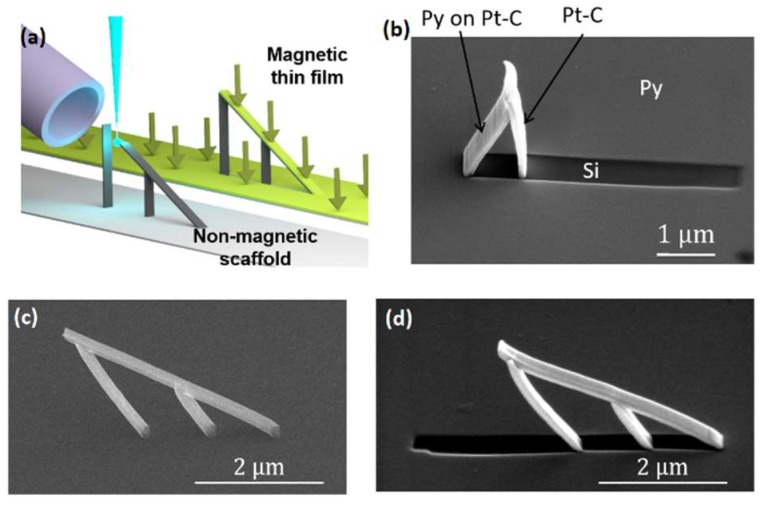
Thermal evaporation of magnetic thin films. (**a**) Process summary: a scaffold is 3D nano-printed (left), followed by the evaporation of a magnetic thin film perpendicularly to the substrate (right); (**b**) SEM image of a 3D nanostructure, result of thermal evaporation at 60° to the substrate; Permalloy (Py) is observed as a light grey layer on top of the Si substrate and the Pt-C scaffold. Evaporation occurred from left-to-right, perpendicular to the nanostructure, casting a shadow onto the Si substrate. (**c**,**d**) 3D nanowire grown at 30° to the substrate before (**c**) and after (**d**) Py evaporation perpendicular to the nanowire, evaporation occurring from right-to-left.

Magnetically-functional 3D nanostructures were achieved by thermally evaporating a thin film (30 to 50 nm) of Permalloy as described in Section 2.3. Thermal evaporation was performed perpendicularly to either the nanostructures or to the substrate, by using the appropriate angle wedge (Figure 5b,c). Figure 9a summarises the 2-step fabrication process followed, in the case of evaporation perpendicular to the substrate. Figure 9b displays a wide ramp, fabricated at 60° to the substrate, which has been evaporated perpendicularly to the ramp.

Different regions, resulting from the strong directionality of the thermal evaporation process, are clearly identified: the Silicon (Si) substrate is covered with Permalloy (Py) everywhere, except where the nanostructure has cast a shadow, where the Si remains uncovered. Evaporation took place from left-to-right in Figure 9b, depositing a film on the left of the Pt-C scaffold. Figure 9c,d display a second nanostructure before (Figure 9c) and after (Figure 9d) thermal evaporation. In this case thermal evaporation was performed from right-to-left in Figure 9d, perpendicularly to the nanostructure, resulting in a smaller shadow since the inclination of the nanostructure in Figure 9d (30°) is smaller than in Figure 9b (60°).

**Figure 10:**
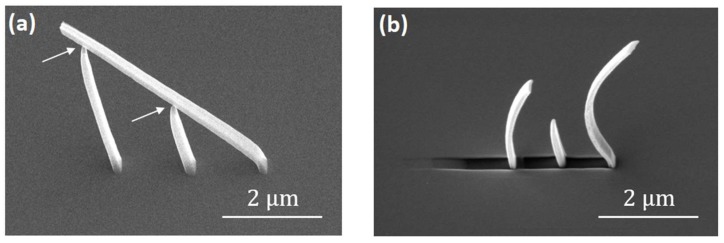
Nanostructure deformation upon thermal evaporation. (**a**) Example structure in which support legs do not fully contact the 3D nanowire, as highlighted by the arrows; (**b**) The same structure after thermal evaporation of a 50 nm Permalloy film.

An important outcome of these thin-film evaporation experiments has been the identification of the need to protect nanostructures against deformation during evaporation. This is illustrated in Figure 10, where we display a nanostructure whose legs did not fully connect to the ramp (Figure 10a). Upon thermal evaporation this structure suffers significant deformation (Figure 10b), which may be caused by strain buildup at the Permalloy/PtC interface, characteristic of physical evaporation processes [19]. It is therefore of great importance to correctly design support scaffolds around 3D nanostructures to minimize scaffold deformation in this kind of application.

### 3.4. Tailored Design for Magneto-Optical Detection

**Figure 11:**
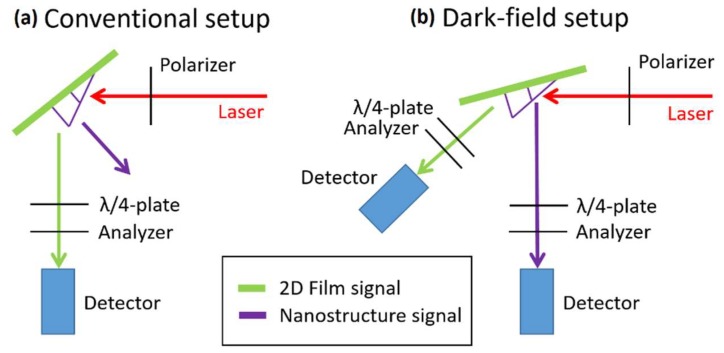
Conventional (**a**) and Dark-Field (**b**) Magneto Optical Kerr Effect (MOKE) magnetometry. A linearly polarized laser (red) is reflected off a substrate, and changes in polarization are analyzed using a combination of a quarter waveplate and an analyzer. In a conventional setup, only the reflection from the substrate is collected (green), while in the dark-field configuration we have recently developed, reflections from both substrate (green) and nanostructure (purple) are analyzed.

3D nanowire structures such as those presented in Figure 9 can sustain controllable 3D transfer of magnetic information, as demonstrated in a recent study [11]. The detection of a single 3D nanowire, necessary to demonstrate such functionality, poses however, a remarkable experimental challenge. Magneto Optical Kerr Effect (MOKE) has been previously used [20] to probe 3D FEBID nanostructures in standard MOKE configuration (Figure 11a). However, in the case of the structures presented in this work, standard MOKE is not a viable technique as the evaporated 2D film of magnetic material would dominate fully the magneto-optical signal [11]. In order to overcome this, the Dark-Field MOKE technique has been developed [11], as shown in Figure 11b. In this variation of MOKE, the angle formed by the nanostructure under study is precisely matched to an optical setup. In the past, Diffraction (or Bragg) MOKE experiments have relied on capturing off-specular reflections due to interference effects in arrays [21,22]. In contrast to this technique, Dark-Field MOKE relies on detecting a specular reflection, coming from a single nanowire. This is achieved by a dedicated optical detector not sensitive to specular reflections from the substrate, due to the relative inclination of nanowire and substrate. It should be noticed that the great control achieved over 3D geometries using the 3D nano-printing methods explained here was key to the success of Dark-Field MOKE experiments.

**Figure 12:**
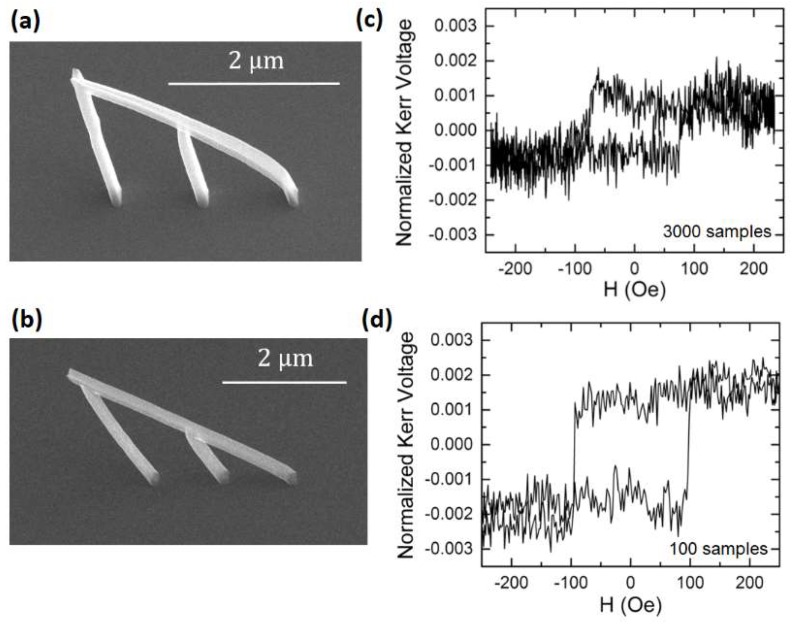
Detectability vs nanostructure quality. (**a**) Nanostructure showing a slight curvature along the ramp, leading to a dispersion of reflection angles; (**b**) Straight nanostructure well matched to the Dark-Field MOKE Setup; (**c**) MOKE loop obtained after 3000 averages at a field sweep rate of 23 Hz for the structure shown in (**a**); (**d**) MOKE loop obtained after 100 averages at a field sweep rate of 3 Hz for the structure shown in (**b**).

As schematically presented in Figure 11b, the dark-field magneto-optical system we have developed uses a single laser (red) in combination with two detection arms (green, purple) to independently measure the magneto-optical response of the substrate and the nanowire under study. The technique relies on specular reflections to collect the light reflected from the nanostructure into the right optical path. It is therefore of great importance to precisely match the nanostructure angle to the configuration of the experimental optical setup, as illustrated in Figure 12. Here, two nanostructures, one (Figure 12a) with a slight curvature along the ramp, and a second one (Figure 12b) straight and well matched to the dark-field setup, are investigated. Figure 12c,d show the corresponding MOKE hysteresis loops, where clear differences in detectability are observed: a MOKE loop with significantly lower signal-to-noise ratio is obtained for the curved nanowire, in comparison with the one with the well-matched straight geometry. This is a consequence of the dispersion in reflected angles created by a curved geometry, leading to substantially noisier loops, in spite of averaging the detected signal a larger number of times.

**Figure 13:**
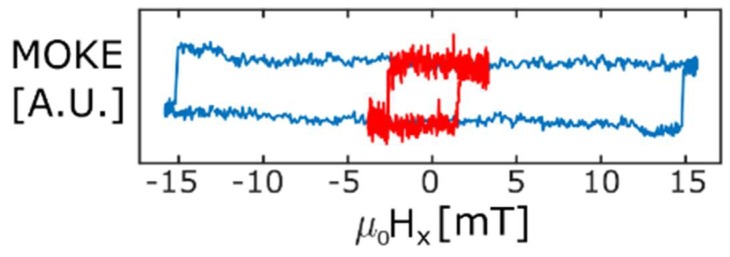
MOKE loops obtained for two rotating fields of different magnitude: 16 mT (blue) and 3.4 mT (red) displaying characteristic signatures of high-quality domain wall transport such as reduction in coercivity and emergence of asymmetries at small field magnitude.

In order to achieve a good control and repeatability over growth, nanostructures precisely matching the dark-field optical setup were grown applying a substrate tilt of 30°, as discussed in Section 3.2. In [11], similar nanostructures were extensively investigated, showing how nucleation, propagation and transmission (depinning from the substrate-wire interconnect) fields, and their spatial symmetries, could be extracted from switching field maps. This allowed us to control the 3D injection and transport of magnetic domain walls in this type of nanowires. Following the same type of methodology, Figure 13 shows two hysteresis loops corresponding to the magnetic switching of a 3D nanowire, under rotating fields with magnitudes larger than the nanowire nucleation field (blue), and close to its transmission field (red). The great difference between switching fields for both and presence of geometrical bias are key signatures of good domain wall transport.

In blue, a large rotating field (16 mT) is applied along the plane parallel to the nanowire direction and perpendicular to the substrate. This results in a hysteresis loop with a large coercivity when magneto optical response is plotted against the projection of the rotating field along the nanostructure (Hx). In red, the magnitude of the rotating field is reduced to 3.4 mT (a field well below the coercivity measured under large rotating fields), and a low-coercivity asymmetric loop is obtained. This is a characteristic signature of systems where domain-wall assisted switching is supported at low fields, but inhibited at larger fields where intrinsic nucleation is more favourable [11,23]. Together with a more detailed study of the 3D susceptibility of domain wall transport and a demonstration of information transfer control [11], these results confirm the capability of the above techniques to produce high-quality and functional magnetic nanostructures for, amongst others, domain wall applications.

## 4. Discussion

A dual-pitch strategy has been employed to extend the capabilities of FEBID 3D nano-printing beyond the growth of thin segment arrays. The possibility of combining new materials by thermal evaporation onto 3D nano-printed scaffolds has also been demonstrated, highlighting the need to employ scaffold supports to prevent structure deformation. These two advancements have been combined with the use of substrate tilt during growth to fabricate and precisely match the geometry of 3D magnetic nanowires to a dark-field magneto-optical setup. The high degree of control and repeatability here demonstrated has enabled the study and control of magnetic information flow in 3D in the form of domain walls, consolidating the role of 3D nano-printing as key tool for rapid prototyping at the nanoscale.

## Notice of Copyright

This manuscript has been authored by UT-Battelle, LLC under Contract No. DE-AC05-00OR22725 with the U.S. Department of Energy. The United States Government retains and the publisher, by accepting the article for publication, acknowledges that the United States Government retains a non-exclusive, paid-up, irrevocable, worldwide license to publish or reproduce the published form of this manuscript, or allow others to do so, for United States Government purposes. The Department of Energy will provide public access to these results of federally sponsored research in accordance with the DOE Public Access Plan (http://energy.gov/downloads/doe-public-access-plan).

## Metadata

In order to comply with EPSRC policy, the metadata associated to this publication can be found at https://doi.org/10.17863/CAM.24917.
